# Supplementary material for: Death in People with Down syndrome: Mortality statistics and novel predictors in US Medicaid and Medicare enrolled adults
Source: medRxiv. 2026 Jul 20:2026.07.17.26358090. Preprint. [Version 1] doi: 10.64898/2026.07.17.26358090 (PMC13419647; doi:10.64898/2026.07.17.26358090)

**Supplement**

Supplement 1: Condition groupings

1. Mental Health: Depression, Attention-deficit/hyperactivity disorder (ADHD), Schizophrenia, Post-traumatic stress disorder (PTSD), Depression, Bipolar disorder, Anxiety disorder
2. Cardiovascular Disease (CVD): Hyperlipidemia, Heart failure, Hypertension, Ischemic heart disease, Stroke, Peripheral vascular disease
3. Cancer: Leukemia, Breast cancer, Colorectal cancer, Prostate cancer, Lung cancer, Any cancer
4. Bone Break: Ankle fracture, Wrist fracture, Tibia/fibula fracture, Femur fracture, Hip fracture, Shoulder fracture, Vertebral fracture, Hip or pelvic fracture

Supplement eFigure1: Positive predictive value of Mortality Risk


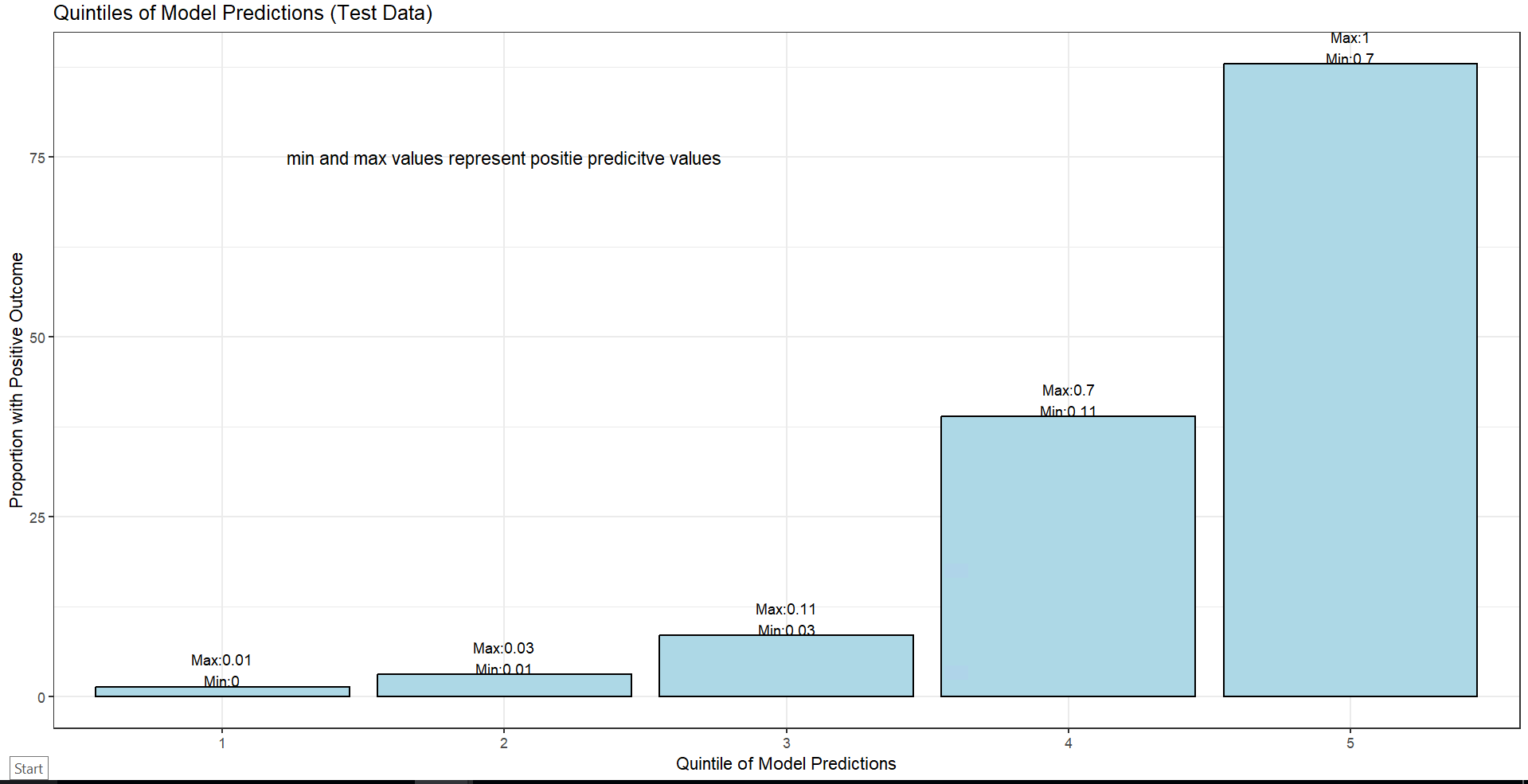


Supplement eFigure2: Model Comparison (XGBoost, Logistic Regression, ElasticNet)


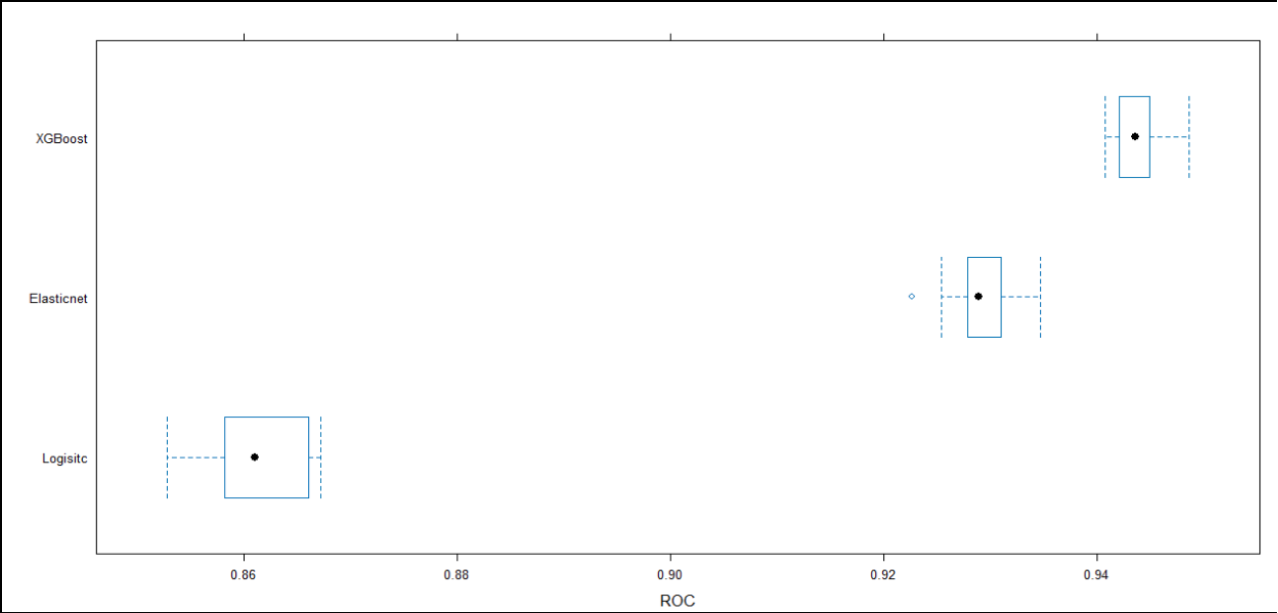

Supplement: Supplement 1 [file media-1.docx]
